# Effects of Background Music on Mental Fatigue in Steady-State Visually Evoked Potential-Based BCIs

**DOI:** 10.3390/healthcare11071014

**Published:** 2023-04-02

**Authors:** Shouwei Gao, Kang Zhou, Jun Zhang, Yi Cheng, Shujun Mao

**Affiliations:** School of Mechanical and Electrical Engineering and Automation, Shanghai University, Shanghai 200444, China

**Keywords:** steady-state visual evoked potential (SSVEP), electroencephalogram, background music, mental fatigue

## Abstract

As a widely used brain–computer interface (BCI) paradigm, steady-state visually evoked potential (SSVEP)-based BCIs have the advantages of high information transfer rates, high tolerance for artifacts, and robust performance across diverse users. However, the incidence of mental fatigue from prolonged, repetitive stimulation is a critical issue for SSVEP-based BCIs. Music is often used as a convenient, non-invasive means of relieving mental fatigue. This study investigates the compensatory effect of music on mental fatigue through the introduction of different modes of background music in long-duration, SSVEP-BCI tasks. Changes in electroencephalography power index, SSVEP amplitude, and signal-to-noise ratio were used to assess participants’ mental fatigue. The study’s results show that the introduction of exciting background music to the SSVEP-BCI task was effective in relieving participants’ mental fatigue. In addition, for continuous SSVEP-BCI tasks, a combination of musical modes that used soothing background music during the rest interval phase proved more effective in reducing users’ mental fatigue. This suggests that background music can provide a practical solution for long-duration SSVEP-based BCI implementation.

## 1. Introduction

A brain–computer interface is a communication system between a human and a computer [1] that allows one to send information or commands from the human brain to the outside world without the need for peripheral neural and muscular activity. Brain–computer interfaces (BCIs) help users express thoughts and control external devices by monitoring their brain activity [2,3]. A variety of methods for monitoring brain activity can be used to acquire brain signals in BCI systems, including electroencephalography (EEG), functional near-infrared spectroscopy [4,5,6], functional magnetic resonance imaging [7], and magnetoencephalography [8]. Among these, EEG has been widely used in practical BCI applications due to its high resolution, convenience of use, and relatively low cost. Among the various types of EEG-based BCIs available, the steady-state visually evoked potential (SSVEP)-based BCI paradigm has received widespread attention due to its high information transfer rate, signal-to-noise ratio (SNR), artifact tolerance and short user training time [9,10]. Generally, the SSVEP-based BCI paradigm requires users to focus more attention on the stimulus target to obtain a high-quality signal. However, the prolonged repetitive stimulation of SSVEP-based BCIs can leave users susceptible to high mental load, which can lead to mental fatigue. Mental fatigue induced by high mental load is usually associated with exhaustion and can severely deteriorate the signal quality of the SSVEP, resulting in decreased task performance [11,12]. Mental fatigue can leave users prone to distraction, increasing the amount of attention required to perform tasks [13]. It has been shown that the amplitude and SNR of signals in SSVEP-based BCIs are related to the mental fatigue of the user and decrease with increasing mental fatigue [14].

Mental fatigue refers to a personal feeling of mental exhaustion after prolonged, high-intensity mental activities, resulting in a decline in behavioral performance [15,16]. To assess mental fatigue in SSVEP-BCI tasks, EEG power can be used to distinguish between different levels of mental fatigue. Activity in the *θ* band of EEG power is mainly associated with sleepiness and cognitive processing, whereas the *α* band occurs mainly in relaxed, lower-attention, and drowsy but clear-headed states. In general, increased mental fatigue is usually associated with an overall increase in EEG power in the *α* and *θ* bands [17,18]. Thus, global EEG activity in the *θ* and *α* bands can be used to assess mental fatigue in SSVEP-BCI tasks.

Mental fatigue and its impact on the long-duration use of SSVEP-based BCIs has long been noted in the literature. Commonly used methods for reducing mental fatigue involve the use of relatively comfortable stimuli, including amplitude-modulated stimulation [19], high-frequency stimulation [20], high-duty cycle stimulation [21], and image-based stimulation [22]. Alternatively, some special stimulus patterns, such as red–green color stimulus patterns and visual noise modulation patterns, have been used [23,24]. However, while most current studies focus on improving stimulation parameters or efficiency, few fundamentally solve the problem at hand (i.e., relieving fatigue and enhancing SSVEP signal through physiological activation). Due to its convenient and non-invasive nature, musical stimulation has been widely used in the scientific community to alleviate mental fatigue. Musical stimulation affects cognition, and listening to music during long periods of high-intensity mental activity can help relieve mental fatigue [25]. Some studies have shown that introducing background music can effectively maintain task performance during periods of prolonged brain activity [26]. In response, some researchers have begun to experiment with the use of background music in BCIs to positive effect [27,28].

Given that background music has been shown to relieve mental fatigue induced by prolonged mental activity and is strongly associated with arousal and attention, this study investigates the introduction of background music in SSVEP-BCI tasks to relieve mental fatigue and improve task performance. The effects of background music on users’ mental fatigue may depend on different factors, and different types of background music may achieve different effects. Therefore, this paper divides music into soothing and exciting styles to study the effects of background music on mental fatigue in SSVEP-BCI tasks and will evaluate changes in SSVEP amplitude, SNR, and EEG power index.

## 2. Materials and Methods

### 2.1. Participants

Six healthy volunteers (four male and two female) aged between 20 and 25 with normal hearing and normal (or corrected) vision participated in this experiment. According to volunteers’ self-reports, none had psychiatric disorders or had taken drugs affecting the central nervous system. Before the start of each experiment, volunteers were asked to get enough sleep, to not exercise strenuously, and to not consume coffee, nicotine, or any other substance that could affect the central nervous system. All participants were informed of the entire experimental procedure and signed informed consent forms as required. All experimental procedures in this study were developed according to the Declaration of Helsinki (1964).

### 2.2. Stimulation Designs

**Figure 1:**
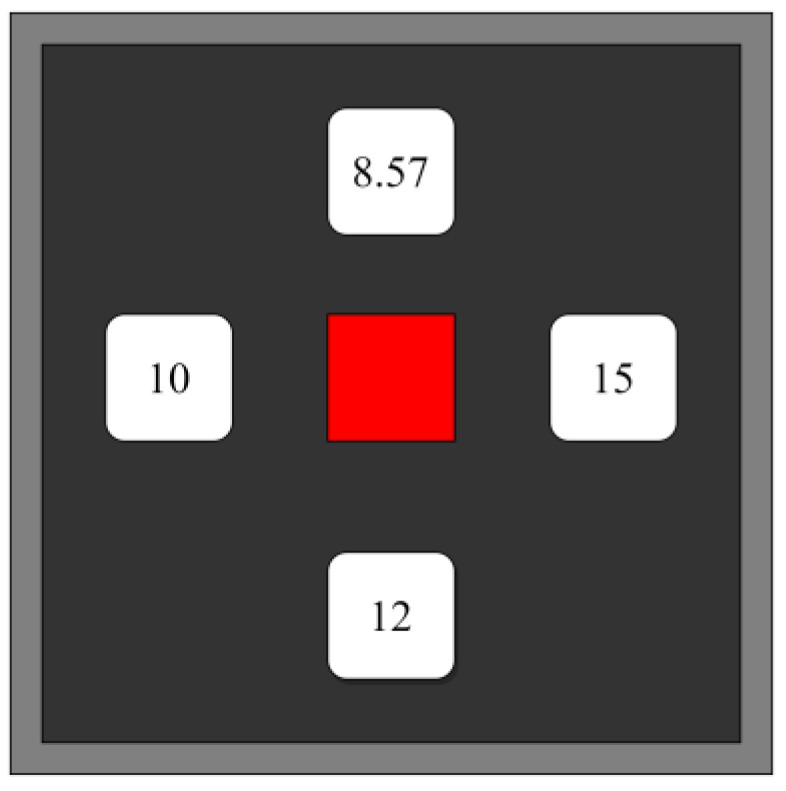
The distribution of the four stimulus targets on the graphical user interface is shown. Each stimulus target has a different frequency.

As shown in Figure 1, four stimulus targets were presented simultaneously to study subjects on a 23-inch LCD monitor with a resolution of 1920 × 1080 at a refresh rate of 60 Hz. Different stimulation frequencies on each target were set (top: 8.57 Hz; left: 10 Hz; right: 15 Hz; and bottom: 12 Hz). In order to avoid harmonic interference between different scintillation frequencies, the multiplier frequencies of each other are not selected here. A red square block in the center of the screen was used as the display in the flashing stop state. The experiments were conducted in a well-lit environment without noise, and each subject sat in a comfortable position 70 cm from the screen, with the center of the screen at eye level. Subjects were instructed to focus their attention on a target stimulus with a frequency of 12 Hz (staring at target stimulus) throughout the experiment. The presentation of all target stimuli in the stimulus paradigm was controlled by PsychoPy software for implementation (https://psychopy.org/, accessed on 10 February 2023).

### 2.3. Experimental Procedure

#### 2.3.1. Procedure of the Main Experiment

**Figure 2:**
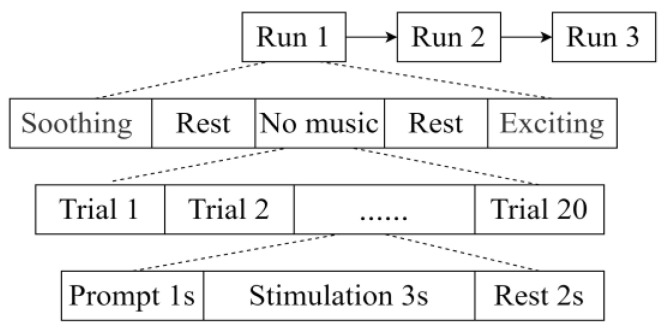
Timing of the main experimental sequence. For each participant, the experimental task was repeated three times.

The time series of the main experiment is shown in Figure 2. Participants were asked to complete three runs of each experimental task. During each run, one of three modes of background music, exciting, soothing, or none (i.e., the control), was randomly presented to each participant. The experimental task was repeated three times. Each run consisted of 20 stimulus units, each lasting 3 s, with a fixed interstimulus interval (ISI) of 2 s. Before each stimulus, a one-second red cue square was displayed on the designated stimulus target to prompt participants to prepare for the stimulus. Participants were asked not to blink or move their bodies during stimulation to reduce the interference of electrooculographic artifacts. To avoid the influence of lyrics on subjects, the materials chosen for this paper (three pieces each of exciting and soothing music) were pure music. To ensure that the volume of the music was constant, music software was used to set the volume to 65 dB. Participants were required to wear Bluetooth noise-cancelling headphones (Sony WH-1000XM4) throughout the experiment.

#### 2.3.2. Extended Experiment

**Figure 3:**
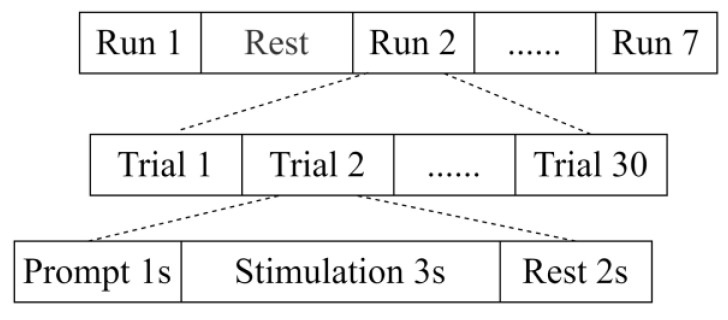
Extended experiment timing diagram.

The time sequences of the extended experiments are shown in Figure 3. The whole experimental task consisted of seven runs and six rest intervals. Each run consisted of 30 stimulation units of 3 s each. Preparation time was 1 s, and the ISI was fixed at 2 s. During the seven rounds of the experiment, subjects performed the stimulus task under exciting background music. There was a 120 s rest interval between every two experimental runs. The six rest intervals were divided into two groups, and three types of background music were performed randomly in each group. The musical materials used in the extended experiment were the same as those used in the main experiment.

### 2.4. Data Recording and Processing

In this study, an Ag/Cl electrode in the Oz position (located at the center of the occipital lobe) served as the input channel. A forehead electrode (Fpz) and left mastoid electrode (A1) were selected as the reference electrode and right leg drive electrode, respectively, and the contact impedance between all electrodes and the skin was kept below 10 K. The sampling frequency of the EEG signal was set to 250 Hz. The EEG signal was amplified by an amplifier and passed through a 50 Hz trap filter to remove industrial frequency interference. Pre-processing of the acquired EEG signal involved bandpass filtering of 0.4–60 Hz using a fourth-order Butterworth filter to eliminate high-frequency interference and low-frequency drift.

#### 2.4.1. SSVEP Amplitude and SNR

Since the frequency of the target stimulus was known, the amplitude spectrum of the SSVEP at the Oz channel for the data segment of length 3 s was calculated by fast Fourier transform. Since the data length was 3 s, the frequency resolution in the amplitude spectrum was 0.33 Hz. In a visual BCI system, the SNR is an important indicator of signal quality. In this study, it was defined as the ratio of the amplitude at the target stimulus frequency to the average amplitude of the six neighboring points:

(1) $$SNR=\frac{6\times y(f)}{\sum_{k=1}^{n/2}\left[y(f+\Delta f\times k)+y(f-\Delta f\times k)\right]}$$

where *y*(*f*) denotes the amplitude of the target frequency in the amplitude spectrum and Δ*f* denotes the frequency resolution.

#### 2.4.2. Evoked EEG Rhythm

To investigate the effects of different types of background music on participants’ mental fatigue in SSVEP-BCI tasks, the EEG power index *α* + *θ* was primarily used to evaluate participants’ mental fatigue levels. To avoid the overlap of the *α*-band rhythm (8–13 Hz) with the target stimulus at 12 Hz, the SSVEP component evoked at the target stimulus frequency was filtered out here using a bandstop filter with 12 Hz. In this study, the power density spectra of *α* and *θ* bands were estimated by the Welch method, and the sum of the band PSD amplitudes on the Welch power spectrum was defined as the EEG power index of *α* and *θ* bands [13].

### 2.5. Statistical Analysis

Since the main experiment was run with a total of 20 stimulus sequences, all stimulus units were classified as fatigue states associated with the timeline. We use the total average value of each index under 1–5, 6–10, 11–15, and 16–20 trials in each run to correspond to four fatigue stages, among which fatigue stage 1 is the state with the lowest degree of fatigue, and fatigue stage 4 is the state with the highest degree of fatigue. To compare the differences in SSVEP amplitude, SNR, and EEG power index between fatigue states 1 through 4 under different modes of background music, a statistical analysis was performed using an Analysis of Variance (ANOVA) and the Bonferroni correction. All data analyses were calculated using SPSS 26 statistical software, and results were considered significant at *p* < 0.05. When the experimental results did not conform to the spherical hypothesis, they were corrected by the Greenhouse–Geisser method.

## 3. Results

### 3.1. Results of the Main Experiment

The mental fatigue states of participants during SSVEP-BCI tasks performed with different patterns of background music was the main object of interest in this study. According to previous studies, participants experience differing degrees of mental fatigue during SSVEP-BCI tasks of long duration, affecting SSVEP amplitudes, SNRs, and the EEG power index.

**Figure 4:**
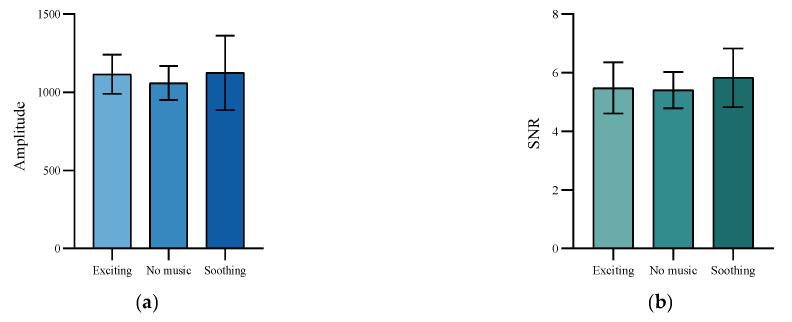

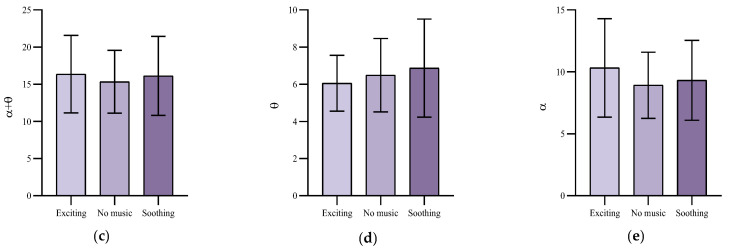
Comparison of the indicators in fatigue stage 1 under different background music modes. (**a**) Amplitude. (**b**) SNR. (**c**) *α* + *θ* band index. (**d**) *θ* band. (**e**) *α* band.

Since the order of presentation of musical modes under each experimental task was randomized, we assumed that the state of mental fatigue in fatigue stage 1 was the same for each participant under different musical modes. To test this hypothesis, a one-way ANOVA was used to assess the differences in initial fatigue among the six subjects in each musical mode during fatigue stage 1. As shown in Figure 4, the amplitude (*F* = 0.53, *p* = 0.60), SNR (*F* = 1.02, *p* = 0.38), *α* (*F* = 0.63, *p* = 0.54), *θ* (*F* = 0.44, *p* = 0.66), and *α* + *θ* (*F* = 0.25, *p* = 0.78) of different musical modes in fatigue stage 1 did not differ significantly, indicating that our hypothesis is correct. To facilitate a comparison of the change in trend of participants’ mental fatigue under different background music modes, we normalized indicators in fatigue stage 4 under different musical modes according to the values in fatigue stage 1 under the corresponding run.

**Figure 5:**
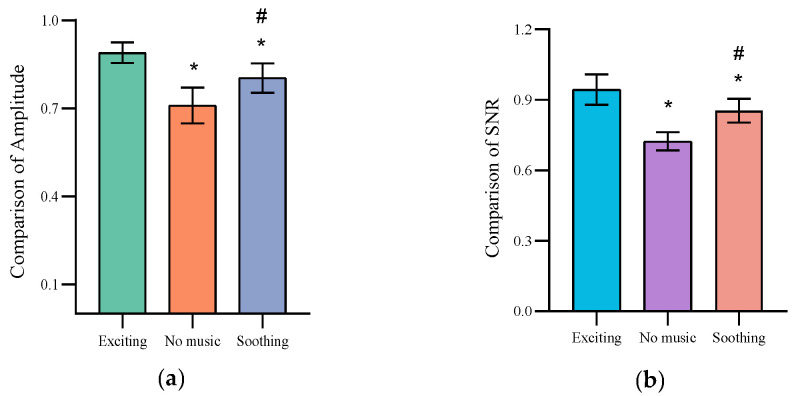
Comparison of the variation of SSVEP amplitude and SNR under different background music modes. (**a**) Comparison of Amplitude. (**b**) Comparison of SNR. *: Significant difference from exciting Music mode. #: Significant difference from no Music mode.

Figure 5 depicts changes in SSVEP amplitude and SNR with different modes of background music for all participants during fatigue stage 4. Compared with fatigue stage 1, the SSVEP amplitudes of fatigue stage 4 in different background music modes exhibited varying degrees of a decreasing trend, as shown in Figure 5a. This is consistent with previous findings that participants experienced mental fatigue with prolonged BCI usage, resulting in decreased BCI performance and lower SSVEP amplitudes [23]. However, SSVEP amplitude trends varied against different background music modes. ANOVA results showed that background music significantly affected SSVEP amplitude change (*F* = 16.38, *p* < 0.05). A post hoc analysis by the Bonferroni method found that exciting background music exhibited the slowest decrease in SSVEP amplitude and better retention. Meanwhile, SSVEP amplitude decreased fastest in the no-music mode, while amplitude during the soothing background music mode fell in between.

A one-way ANOVA was also used to analyze the SNR trend of the SSVEP in different background music modes under fatigue stage 4. The results show that the SNR exhibited a trend similar to that of amplitude in fatigue stage 4 (*F* = 22.2, *p* < 0.05). As shown in Figure 5b, after a long-duration BCI task, the SNR of the SSVEP in the exciting background music mode showed the smallest overall change compared with the soothing and no-music modes. These results suggest that the introduction of background music could alleviate the deterioration of signal quality caused by mental fatigue during SSVEP-BCI tasks, with exciting background music showing the greatest anti-fatigue effect.

**Figure 6:**
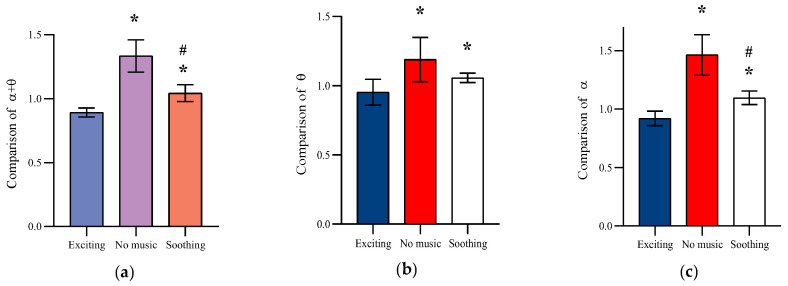
Comparison of the variation of *α*, *θ*, and *α* + *θ* under different background music modes. (**a**) Comparison of *α* + *θ*. (**b**) Comparison of *θ*. (**c**) Comparison of *α*. *: Significant difference from exciting Music mode. #: Significant difference from no Music mode.

The extent of mental fatigue was mainly reflected in the trend of participants’ EEG power index *α* + *θ*. The normalized EEG power index for the six participants is shown in Figure 6. A one-way ANOVA found different background music mode to have a significant effect on the change in the *α* + *θ* power index (*F* = 35.09, *p* < 0.05). During the SSVEP-BCI task, participants in the no music mode had a significant increasing trend in the *α* + *θ* power index under fatigue phase 4, which responded to a continuous increase in participants’ mental fatigue. Meanwhile, the *α* + *θ* power index decreased in both exciting and soothing musical modes, with the most significant decrease observed in the exciting mode. Similar trends in *θ*-band (*F* = 7.11, *p* < 0.05) and *α*-band (*F* = 37.09, *p* < 0.05) power indices were observed for different background music modes, with exciting music modes showing significant improvement at the *θ* and *α* power indices compared with the no-music mode.

### 3.2. Results of the Extended Experiment

**Figure 7:**
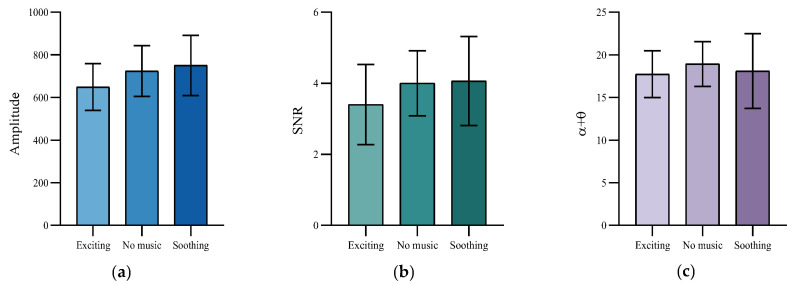
Comparison of the indicators in pre-intervention state under different background music modes. (**a**) Amplitude. (**b**) SNR. (**c**) *α* + *θ* band index.

This is an extended experiment based on the main experiment. To reflect the intervention effects of different musical modes on participants during the rest interval, we used the last five target stimuli of the experimental run before each rest interval as the pre-intervention fatigue state, and we used the first five target stimuli of the experimental run after the rest interval as the post-intervention recovery state. As shown in Figure 7, a one-way ANOVA of the pre-intervention indices of different music modes in the two group rest intervals showed that no significant differences were found in amplitude (*F* = 1.07, *p* = 0.37), SNR (*F* =0.65, *p* = 0.53), or EEG (*F* = 0.20, *p* = 0.82) power index, and participants’ fatigue status before intervention was approximately the same in different musical modes. This may be due to the fact that experimental conditions were consistent for participants during the run of the experiment prior to the rest interval. For later analysis, the mean values of SSVEP amplitude, SNR, and EEG power after intervention in different musical modes were normalized to the values corresponding to the pre-intervention state.

**Figure 8:**
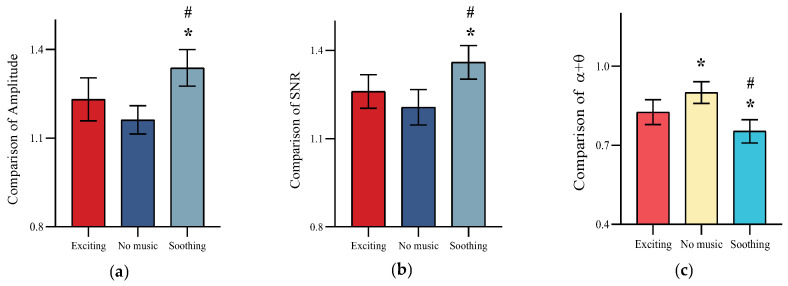
Comparison of variations in SSVEP amplitude, SNR, and EEG power index *α* + *θ* under different background music modes. (**a**) Comparison of Amplitude. (**b**) Comparison of SNR. (**c**) Comparison of *α* + *θ*. *: Significant difference from exciting Music mode. #: Significant difference from no Music mode.

As shown in Figure 8, the signal quality of the BCI system is improved in the extended experiment, as both the magnitude and SNR of the SSVEP show an increase after the rest interval adjustment. However, SSVEP amplitude (*F* = 12.42, *p* < 0.05) and SNR (*F* = 10.70, *p* < 0.05) trends varied for different musical modes. The rest interval phase of soothing background music had the greatest effect on enhancing SSVEP amplitude and SNR. At the same time, an ANOVA (*F* = 16.56, *p* < 0.05) was conducted on the *α* + *θ* power index for different combinations of musical patterns, and it was found that the soothing background music mode provided the most significant relief of participants’ mental fatigue.

## 4. Discussion

According to studies on mental fatigue in SSVEP-based BCIs, mental fatigue causes a decrease in the user’s arousal level and performance, which leads to a decrease in the performance of BCI tasks. Increased mental fatigue also leads to user drowsiness and distraction, which in turn contributes to an increase in the mental effort required to maintain attention during SSVEP-BCI tasks, thus exacerbating fatigue. In this paper, we contend that the mental fatigue brought on by SSVEP-BCI tasks can be effectively relieved by the introduction of background music. We used the SSVEP amplitude, SNR, and EEG power index to compare mental fatigue and the anti-fatigue performance of SSVEP-BCI tasks against different background music modes. More specifically, decreases in attention, arousal levels, and task completion ability were associated with increases in global EEG power in the *θ* and *α* bands [13,29]. According to previous fatigue studies, the *θ* band was associated with fatigue, working memory, and changes in mental state [30]. Participants who felt mental fatigue during the experiment experienced drowsiness as well as a decrease in task completion ability and, thus, an increase in *θ*-band power. In addition, fatigue-related increases in the *α* band are also closely associated with efforts to maintain vigilance levels [31,32], with the *α* band occurring mainly in relaxed, alert, or mildly drowsy states. When participants experience mental fatigue, their levels of attention and alert decrease. Therefore, compared to the alert state, participants need to devote more focused attention, increase mental effort to focus on the target, and maintain alertness levels during the fatigued state. Finally, increased mental fatigue in participants was also associated with a reduction in SSVEP amplitude and SNR, with ripple effects on BCI performance.

This paper’s main research objective was to investigate the effects of three modes of background music on mental fatigue induced by long-duration SSVEP-BCI tasks. In the no-music mode, we found an expected rise in *θ*-band, *α*-band, and *α* + *θ* powers with increasing mental fatigue, a decreasing trend in the amplitude and SNR of the SSVEP, and a significant deterioration in the signal quality of the BCI. Meanwhile, for BCI tasks conducted in both musical modes, SSVEP amplitude, SNR, and EEG power benefited from the effects of background music, with significant improvements during long-duration BCI tasks. These findings suggest that background music has an arousing effect on participants’ attention and may provide a degree of compensation for mental fatigue during BCI tasks. A detailed analysis of the results showed that participants in the exciting musical mode were more alert, exhibited the lowest levels of mental fatigue, and had the best performance on the SSVEP-based BCI task. Therefore, we recommend introducing exciting background music during long-duration SSVEP-BCI tasks for better BCI performance.

Researchers generally agree that when mental fatigue occurs during long-duration cognitive tasks, performance declines primarily as a result of a decrease in the attentional resources allocatable to the task at hand [33,34,35]. Among the brain areas responsible for allocating resources, the anterior cingulate cortex, which is associated with assessing rewards [36], appears particularly susceptible to the effects of mental fatigue [37]. A theory in favor of a failure of dopaminergic networks has been proposed by some researchers to explain this phenomenon [38]. According to this theory, mental fatigue causes a decrease in the activity of the central nervous dopamine system. The beneficial effects of background music on mental fatigue may be attributable to its positive influence on dopamine levels [25]. In general, the introduction of background music can effectively relieve the mental fatigue brought on by long-duration BCI engagement, thus improving BCI performance. In addition, participants tend to be more easily motivated by the fast-paced and high-energy character of exciting background music, and this can encourage dopamine secretion and higher levels of attentional arousal. Thus, the compensatory effects of exciting music on mental fatigue during cognitive tasks is also more obvious.

Certain rest intervals usually exist during long-duration, continuous SSVEP-BCI tasks to alleviate the effects of mental fatigue on the individual. The mental load on users during the rest interval phase is relatively low because users do not need to maintain attention for intense cognitive tasks during this phase [18]. We theorized that the effects of background music on users’ mental fatigue might differ under different states. On this basis, we invited the six study participants to an extended experiment, based on the main experimental study in this paper, to investigate the interventional effects of different combinations of background music modes on mental fatigue during a continuous SSVEP-BCI task that included rest intervals. Since mental fatigue is mainly associated with an increase in global EEG power of *θ* and *α* bands [29], it was evaluated here based on SSVEP amplitude, SNR, and EEG power. Expanded experiments showed that soothing background music can play significant role in improving users’ mental fatigue during the rest interval. We speculate that during the lower mental load phase, soothing background music allows participants to rest more fully and recover from mental fatigue more quickly. While exciting background music during the SSVEP-BCI task increased participants’ arousal, soothing background music during the rest interval phase was beneficial for participants’ rapid recovery from mental fatigue. Thus, during continuous SSVEP-BCI tasks, combined modes of background music may achieve better BCI task performance and lower mental fatigue than either mode alone.

## 5. Conclusions

In conclusion, this paper recommends the introduction of background music during SSVEP-BCI tasks to relieve mental fatigue. To demonstrate the feasibility and effectiveness of the proposed method, experiments on SSVEP-BCI tasks using different modes of background music were conducted in this paper. Experimental results consistently showed that the introduction of background music effectively relieved participants’ mental fatigue, and exciting background music improved the performance of SSVEP-BCI tasks. To account for the real-world use of the SSVEP-BCI tasks, this paper also designed extended experiments to investigate the effects of different combinations of background music modes on continuous SSVEP-BCI tasks with rest interval phases. The experimental results show that a combined approach using soothing background music during the rest interval is more conducive to alleviating the mental fatigue of BCI users.

## Data Availability

The data in this study are available from the corresponding author on reasonable request.
